# On Animal Decomposition as the Chief Promotive Cause of Cholera*Abstract of a paper read before the Philadelphia County Medical Society, April, 1855.

**Published:** 1855-08

**Authors:** Henry Hartshorne


### MEDICAL EXAMINER.

NEW SERIES.—NO. CXXVIII.—AUGUST, 1855.

### ORIGINAL COMMUNICATIONS.

### *On Animal Decomposition as the Chief Promotive Cause of Cholera.** By Henry Hartshorne, M.D.

Notwithstanding all that has been said upon the subject, sufficient emphasis does not appear yet to have been placed upon the influence exerted in the propagation of Epidemic Cholera by *animal*, as contrasted with *vegetable* decay and decomposition.

A marked difference evidently exists, in this respect, between Cholera, on the one hand, and, on the other, Autumnal Fever, whose promotive influences are either vegetative or telluric, and Yellow Fever, which seems to require a certain combination of both. In correspondence with this, a striking contrast to yellow fever and its kindred diseases is found in the fact that, in India, Europeans are less subject to the cholera than natives. In the Mauritius, and elsewhere, negro slaves, who are insusceptible of the fevers of the climate, are quite as liable (or from habits of living, more so) as any others, to cholera.†

A few facts may be appropriately given, to demonstrate the position implied in the title of this paper.

*Abstract of a paper read before the Philadelphia County Medical Society, April, 1855.

† Orton on Cholera in India, pp. 450–453.

The Gangetic Delta being the great focus and centre of the disease, certain traits in the usages and circumstances of that locality and its population are found to have a direct bearing upon the subject. Among these may be named, the peculiarity of the inhabitants in disposing of the dead. Only the Mahommedans, in India, and the peers of highest rank, bury their dead. The mass of the people either burn the bodies, or throw them into the water, without any sort of interment. Supposing, even, that the perfect incineration of human corpses would remove all perniciously decomposable matter, there is evidence that the way in which it is done, or not done, must rather aggravate the evil.* I have heard the incompleteness of the burning described by an eye-witness. But large numbers of the poorer classes are constantly being thrown, after death, into the Ganges and its tributaries; that river being held to be a sacred stream —the gateway to heaven. A traveller saw twenty bodies thus disposed of in one morning before breakfast! At Allahabad, at the junction of the Ganges and Jumna, many persons drown themselves, as devotees; and others are often drowned by the pressure of crowds immersing themselves in the sacred spot.† But a still greater mortality attends the worship of Juggernauth; the annual average of pilgrims to whose temple is said to be 120,000. Thousands of these poor wretches die from famine, fatigue and exposure, during the journey, and are left to rot. In half an acre, 150 bodies have been counted.‡ One writer (Asiatic Journal), asserts that he saw 300 unburied bodies within five miles. Lieut. Colonel Forest§ met with "half a mile of human skulls upon a bend of the river."

Add, then, to these facts, that the Ganges yearly overflows its banks, from one to two hundred miles in some places. The efflux of such an inundation, in the dry season, must expose a quantity of piscine and other animal matter to putrefaction, perhaps nowhere else to be seen. From the geological formation of the Delta, Sir Charles Lyell || observes, that "it is easy to per-

* Stocqueler, Handbook of India, p. 366.

† Ibid, p. 407.

‡ Ibid, p. 508.

§ Tour along the Ganges and Jumna.

|| Geology, vol. iv., p. 359.

ceive that both animal and vegetable remains must continually be precipitated into the flood, and sometimes become imbedded in the sediment which subsides in the delta."

S. Rogers, F.R.S., a British army surgeon, in his report on cholera in the Madras army, gives the following kindred facts:—

"The Coom river nearly encircles the village of Chintandrepett. This river was made a privy of by hundreds of natives daily; and when the monsoon was heavy, and the bottom of this Augean stable thoroughly cleansed, no ill resulted, but if the monsoon failed, and the river remained uncleansed, when the hot weather returned, the water became low, and the filth at the bottom was exposed to the sun, the smell was most offensive, and *an attack of the cholera was the certain result*, the only victims being those residing within a short distance from its banks."

In the western part of Europe, influences connected with the living have long been noticed, in closely aggregated communities, to display a similar propagative power.

The facts which the elaborate statistics, researches and arguments of Dr. Baly's report to the Royal College of Physicians, (London, 1854,) have brought to bear upon the opinion that *human intercourse* is, at least, one of the principal modes of transmission and migration of the epidemic cause, have *this* extent precisely. The learned author is barely willing to admit contagion as a rare, contingent *possibility*. It is difficult to evade the admission of this possibility. Certain facts maintain a degree of stubbornness in regard to it, difficult to reason away. I allude to such facts as those quoted by Dr. Alison* as occurring in Edinburgh in 1832, in Toulon, in 1833, and in Arbroath, Scotland, in 1853; those given by Dr. Berg in his treatise on the Cholera in Sweden, in 1850; one instance by Dr. S. H. Dickson, in Charleston, 1832,† and those mentioned by Drs. Simpson and Craig‡ as occurring in Scotland during different years; all cases in which the evidence consisted not merely in a succession of persons having been attacked after contact with the sick, but in that *these persons only* were attacked, for a long time before

* British and Foreign Medico-Chirurg. Review, Jan. 1854.

† American Journal of Medical Science, vol. xiii. p. 309.

‡ Edinburgh Monthly Medical Journal, 1849.

and after, while those around differed in no circumstance, except in the absence of such exposure. Other cases have also been given by Littré, Velpeau, Gendron, &c.* But all considerations enforce the exceptional character of such facts; while examples of the contrary,—that is, of the manifest absence of contagion under the most favorable circumstances for it, are innumerable. As an illustration of these I may name only that of Annan, in Scotland; which is a town nearly equidistant from Carlisle and Dumfries, between which places a constant communication is always kept up via Annan, this being directly in the main line of traffic; and yet this town was entirely exempt from cholera, in 1832 and 1848, although the disease prevailed both in Dumfries and Carlisle.†

We come, then, to find, in the facts and inductions of Dr. Baly, evidence quite in apposition with the present enquiry, the alternative of contagion, as a rule, being withdrawn. It is proper, however, to guard our admission of the force of these inductions, by refusing to believe that human intercourse has been the *only* means by which the epidemic has migrated, even across the ocean. The instances of the Tonawanda and Tuscarora, upon which the cholera broke out after two weeks, more or less, at sea, and in one of which it was cut short by an iceberg, while neither brought any sick into port, are familiar. In these, and many other similar occurrences, the disease is to be accounted for by an atmospheric visitation alone. The efforts made by Dr. Baly to do away with this clear explanation in the case of the two ships New York and Swanton, (December, 1848), are among the least philosophical of his reasonings; importation by them being impossible, as there was no cholera at Havre when they left there, and the passengers, Germans, had been three months domiciliated in the place before they sailed.‡ Dr. Baly does not deny the atmospheric mode of extension as occurring sometimes; although he believes it to be much more often traceable to *infection by means of human intercourse.* As, then, his careful analysis of

* Vide Tardieu on Epidemic Cholera, p. 114.

† Account by Dr. Grieve, of Dumfries, in Edin. Monthly Journ. of Med. Sci., April, 1854.

‡ Report, by Dr. James Wynne, on Cholera in the United States, in 1849 and 1850.

the facts, as well as the aggregate testimony of other observers, proves that this mode of infection does not consist in the *reproduction of the poison in the bodies of the sick*, so as to emanate from them as a true contagion,* we must infer that it is only by affording the conditions for the multiplication of the cause without the bodies of both sick and well, that human intercourse usually has its effect.

The universal report of those who have contributed to the statistics of Dr. Baly, is to the effect that mortality from cholera is almost invariably commensurate with the filth and destitution of the inhabitants and their abodes. Abundance of testimony† is afforded to the same point by other writers, especially in regard to large, crowded, and dirty towns; as in Moscow, Paris, Marseilles, Liverpool, Manchester, Edinburgh, &c. In Moscow, in 1832–33, the deaths were as high as 1 in 32 of the whole population.

In Holland, Suermann found the mortality to be 1·54 in 1000 in rural districts; in the towns, 8·93 in 1000. This latter fact may suggest to our consideration the comparatively slight degree in which lowness and dampness alone affect the mortality from this disease. And the candid assertion of Dr. Baly himself, that some of the districts in which the rate of mortality was highest in Great Britain, in 1849 as well as in 1832, "have a high level, and lie in the central regions of country where the rivers take their rise," may more clearly shew that elevation does not afford immunity, and that lowness and dampness act so far only as they are promotive of the accumulation of animal filth and its products. "In the evidence received," says the same writer, "lowness of site is not very prominently set forth among the unfavorable sanitary conditions; being, in fact, specifically menmentioned only *five* times." Yet out of 68 places where cholera raged, bad ventilation and over-crowding of houses are mentioned *fifty four times*, defective drainage twenty eight times, cess-pools, open sewers, &c., sixteen times, &c. Among the striking instances in which a very partial distribution of the epidemic occurred in almost contiguous localities, *pari passu* with variation in the

* Schmidt, Characteristik der Cholera, p. 81.

† E. g., Milroy, on Cholera and Quarantine; Reid, Letter to Lord Morpeth, 1848; Starr, Discourse on Asiatic Cholera, etc.

above respects, I may remind the reader of that of the three prisons at Wakefield,* on the same plot of ground, seventeen acres in extent.

We may recall here, also, the illustrative fact, that Orton, in his excellent treatise on the Cholera in India, while alluding to the effect of high and dry sites, observes, that "unhappily the immunity arising from situation was only temporary."† Again, Jameson, in a history of the Epidemic Cholera of Bengal,‡ mentions that it would "sometimes single out *elevated* and *airy* points, sparing those in the plain below." "It *always*, however," he adds, "affected a *crowded population.*" In Russia, Dr. Frettenbacher, of Moscow, states, in a statistical report, that "it propagated itself *especially* in unhealthy and confined dwellings."§ S. Rogers, already, quoted, says, expressly, that it "has no (direct) reference to the geological structure of the country."

A valuable amount of testimony is afforded in regard to the influence of the *drinking water* of localities. The sweeping generalization of Dr. Snow, to the effect that this, namely, conveyance by the drink and food of the sick and others, was the principal or only mode|| by which the disease was extended and transported, proves to be incorrect; witness the instances of attack at sea, of slow migration over sea, &c. But the effect of the water-supply is still of great importance, as showing the power of *animal contamination.* Bethlehem Hospital, supplied by an artesian well, had among 400 inmates, no case of cholera in 1849; it being the only large lunatic asylum in London which escaped, as it was the only one furnished with spring water. The facts with regard to the different districts supplied from the Thames, some from above and others from below the entrance of the sewers, are very remarkable; showing a difference, between the two extremes, of a mortality, in the one case, (of contaminated water) of from 28 to 205 in the 10,000 inhabitants, and in the other, (purer water) of only from 8 to 33 in the same number.¶

* Op. citat. p. 20.

† Op. citat. p. 404.

‡ Reviewed in Chapman's Journal, vol ii. p. 361.

§ Gazette Médicale, Jan. 13th, 1849.

|| Oration on Continuous Molecular Changes, p. 18.

¶ Baly's Report, p. 205.

Let us refer, in this connection, to the conclusions reported by Dr. Pettenkoffer, of Munich, to the Bavarian Government.* The substance of them is essentially as follows:

"The products of decomposition of human and animal excrements contained in the soil, appear to be the elements that determine the soil's capability for absorbing the miasm, independently of the elevation above the level of the sea."

"The excrements of cholera patients, when in a state of decomposition, become fertile sources for the propagation of the disease in families."

It is, we need hardly repeat, justly observed by Dr. Baly, that the influence of the drinking or other water cannot be shewn to consist in its serving as a vehicle for a poison, a contagion, generated specially in the bodies of those who have suffered from the disease. We have seen that this cannot be, since there *is no such* contagion, generally speaking, if it even can ever exist. I refer once more, upon this point, to the inquiries and experiments of Schmidt, and of Meyer, Marshall, and others, mentioned by Dr. Gull,† as well as to the earlier ones of Lizars, Coste, &c.,‡ and those of the surgeons and medical students at Moscow and Dantzic, in 1832.||

As illustrative of the same topic, the observations of Prof. Thiersch, of Munich, should be alluded to.§ He found that "the matters evacuated by vomiting and diarrhœa do not, when recent, propagate the disease; they are not contagious. If the matters evacuated by stool are left to themselves, at a temperature of from 41° to 50° Fahr., in from three to seven days a change takes place; they undergo a process of fermentation; they are then capable of exciting cholera in healthy individuals." This position is supported by an account of careful trials made upon animals. These experiments being, of course, performable only at the time and place of the prevalence of a cholera epidemic or endemic, there is nothing to oppose, but everything to favor the opinion, that matters of the kind mentioned serve only, like

* Med. Times and Gazette, Nov. 18th, 1854.

† Reports, &c., p. 122.

‡ On Cholera Asphyxia in 1832, p. 61, &c.

|| Ibid, p. 62, note.

§ Med. Times and Gazette, Nov. 1854.

other decomposable animal materials, as a *pabulum* or *nidus* for the sustenance and development of the unknown *specific cause.*

We may remark, also, that the same explanation would appear to apply to the experiments narrated in the Edinburgh Medical and Surgical Journal (April and October, 1854) by Dr. Lauer Lindsay. These afford us no reason for their exemption from the category in which Dr. Lindsay places those of Mr. Marshall, published a year earlier,* namely, that "all the experiments hitherto made appear to be negative in their results, objectionable, or inconclusive." Referring to the observations of Dr. Thiersch, just given, we may note, upon this point, the fact that the time during which the animals (dogs) used by Dr. Lindsay were exposed to the combined effects of confinement, swallowing and breathing the matters of evacuation and perspiration of cholera patients, was in no case less than seven days (from the 19th to the 26th Nov.) before marked symptoms occurred; and that these symptoms ought hardly to be called *specific*, as the discharges were "decidedly biliary,—green—and greenish feculent matter," and "emitting an intolerable stench." Dog No. 1 is also stated to have become *somewhat lively and greatly better* after having eaten a quantity of the flesh, fat and blood of dog No. 2, which died on the previous day. The expression of Mr. Marshall is clearly correct, that this evidence is still "short of actual proof."

Leaving trans-atlantic authorities, we shall find in the new world a still increasing force in the testimony in regard to the subject of this discussion. On referring to the "Report by James Wynne, M.D., on Cholera in the United States in 1849 and 1850†," we may observe, upon almost *every page*, matter directly to our purpose.

A single quotation may therefore suffice as an example and illustration of this.

"As a *general fact*," says Dr. McPheeters, of St. Louis,‡ "the cholera prevailed most in those parts of the city in which there were the *largest number of persons herded together*, where the streets were unpaved, and where there was the greatest amount of *filth* and moisture." In Louisville, Buffalo, New York,

* Brit. and For. Medico-Chirurg. Rev., April, 1853.

† Presented to both Houses of Parliament, and published in 1852.

‡ Op. citat., p. 15.

Philadelphia, and Boston, essentially identical, and equally emphatic, statements are made by the correspondents and collaborators of Dr. Wynne. In Baltimore, some very striking facts occurred* in the history of the epidemic in the Almshouse, in July, 1849. No cases being in the city at the time, those which took place in that Institution were accounted for (by Dr. Buckler) by the presence of a very large and foul overfloiwng cesspool, whose contents mingled with the washings of the dead house, &c. &c., upon the north side of the building. Upon this side, where there was a door opening to the north, all of the lunatic inmates, seventeen in number, were attacked with cholera, and all died. *The remaining inmates of the same building entirely escaped.* Of the eight medical students attached to the Almshouse, the four whose rooms were not thus exposed, escaped. The manager, who slept in a room above that of the students, looking to the north, was likewise seized with the disease, but recovered. His family, whose rooms looked to the south, escaped. In the cases which occurred among the pauper inmates, those generally were attacked who slept in a position exposing them to the wind from the north.

The whole tenor of the reports, from which the above excerpts are derived, is decidedly adverse to the opinion of *contagion* being at all a frequent, if it be even an occasional mode of propagation of cholera.

In conclusion, I will add only a brief allusion to a few localities and occurrences not mentioned in the above documents.

The history of the fatal epidemic at Columbia, Pa., in September, 1854, is very well known. Cholera had never appeared in that town before. What was the reason of its heavy visitation then? Chiefly the facts, that an exceeding drought had reduced the channel of the river to an unusually low ebb, and that, in its bed, a short space above the town, a number of carcases of sheep and other animals, thrown from the railroad trains, &c., were putrefying rankly in the sun. A reservoir which supplied many of the people with drinking water was filled from the river not far from that spot, and the wind blew from it directly over the town. If we are correctly informed, the first subsidence of the disease attended a change of the wind.

At Pittsburgh, shortly after the above events, a similar epidemic

* Ibid, p. 72.

occurred. A gentleman on a visit to that locality not many days before the disease broke out, informed me that the same condition of the rivers existed there, with a similar abundance of *accumulated, putrefying, animal matter*, exposed to the sun.

In Rhode Island, in the autumn of the same year, the writer was told that the local existence of cholera in a few spots, otherwise very healthy, might be traced in coincidence, at least, with a practice not uncommon along the shore of the sea or bays, of dragging up fish in quantities by nets, and spreading them out to rot for manure.

And, lastly, in Barbadoes, where a considerable fatality from cholera has at different times occurred, Dr. W. H. Freeman, late U. S. Consul to that island, reports a very similar sanitary condition to those above related, even to some extent recalling the last item mentioned.

There may be, perhaps, no actual novelty in the views above expressed, more than in the well-known facts by which it has been endeavored to support them. My object has been, simply, to collate such data as would seem to show the paramount comparative importance of animal matter in a state of "post-organic" change, as the food or fuel of the cholera-cause. The difference between this disease and Typhus (in origin) would appear to be, that while the latter may almost *invariably* be produced by the persistence of certain unfavorable sanitary conditions, the former, Cholera, is generated *only in the presence of a certain unknown contingent*, whose capriciousness of migration, partial subjection to temperature, and other habitudes, suggest the probability of the animalcular hypothesis.

Whatever the theory, the lesson from all the facts is one (often told but not yet well learned) of hygiene and prevention. Cities should be built and regulated to prevent epidemics, as they should be to afford security from conflagrations. The laws of public benevolence, like those of private morality, are an essential part of the economy of the world. As personal vice brings misery, by the violation of physical laws, so the aggregate vice of communities, and the neglect of the higher classes to do their best for those around them, meet with retribution, in those scourges, which, under the forms of plague, cholera, typhus, and yellow fever, desolate populations almost in proportion to the errors of their local life.

